# Time of Default in Tuberculosis Patients on Directly Observed Treatment

**DOI:** 10.4103/0974-777X.68533

**Published:** 2010

**Authors:** Geeta S Pardeshi

**Affiliations:** *Department of Preventive and Social Medicine, Dr. Shankarrao Chavan Government Medical College, Nanded, Maharashtra, India*

**Keywords:** Control, Default, Directly observed treatment–short course (DOTS), Tuberculosis

## Abstract

**Background:**

Default remains an important challenge for the Revised National Tuberculosis Control Programme, which has achieved improved cure rates.

**Objectives:**

This study describes the pattern of time of default in patients on DOTS.

**Settings and Design:**

Tuberculosis Unit in District Tuberculosis Centre, Yavatmal, India; Retrospective cohort study.

**Materials and Methods:**

This analysis was done among the cohort of patients of registered at the Tuberculosis Unit during the year 2004. The time of default was assessed from the tuberculosis register. The sputum smear conversion and treatment outcome were also assessed.

**Statistical Analysis:**

Kaplan-Meier plots and log rank tests.

**Results:**

Overall, the default rate amongst the 716 patients registered at the Tuberculosis Unit was 10.33%. There was a significant difference in the default rate over time between the three DOTS categories (log rank statistic= 15.49, *P*=0.0004). Amongst the 331 smear-positive patients, the cumulative default rates at the end of intensive phase were 4% and 16%; while by end of treatment period, the default rates were 6% and 31% in category I and category II, respectively. A majority of the smear-positive patients in category II belonged to the group ‘treatment after default’ (56/95), and 30% of them defaulted during re-treatment. The sputum smear conversion rate at the end of intensive phase was 84%. Amongst 36 patients without smear conversion at the end of intensive phase, 55% had treatment failure.

**Conclusions:**

Patients defaulting in intensive phase of treatment and without smear conversion at the end of intensive phase should be retrieved on a priority basis. Default constitutes not only a major reason for patients needing re-treatment but also a risk for repeated default.

## INTRODUCTION

Tuberculosis remains a worldwide public health problem, with India accounting for nearly one fifth of the global burden of tuberculosis. The services of Revised National Tuberculosis Control Programme (RNTCP) under the DOTS (Directly Observed Treatment–Short course) strategy has been made available in the entire country by March 2006. RNTCP has achieved improved cure rates and reduction in unfavorable outcomes.

Default is one of the unfavorable outcomes for patients on DOTS and represents an important challenge for the control program. Inadequate treatment adherence is considered as a potential cause of drug resistance.[1] Studies in India and other developing countries have focused on various causes and risk factors for default. Gender, alcoholism, treatment after default, poor knowledge of tuberculosis, irregular treatment and socioeconomic status are some of the factors which have been found to be associated with higher default rates.[2–5] Other factors related to the disease, patients and service providers have also been identified as reasons for noncompletion of treatment.[6–10] Yet it has remained difficult to predict non-adherence to treatment.

This study throws light on a particular aspect of default, i.e., the time of default after initiation of treatment.

## SUBJECTS AND METHODS

RNTCP was initiated in Yavatmal district, Maharashtra, India, in August 2002. By the end of December 2004, the entire district was covered under DOTS. The district has an annualized total case detection rate of 134 per lakh of population and a cure rate of 82% in smear-positive patients.[11]

This analysis was done on a cohort of patients registered for DOTS at the Tuberculosis Unit (TU) in the District Tuberculosis Centre (DTC), Yavatmal, from 1^st^ January 2004 to 31^st^ December 2004. The population covered by this TU was 577,398. The data regarding DOTS category, smear conversion at the end of intensive phase; treatment outcome; and time of outcome were collected from the tuberculosis register at the DTC. As secondary data was collected and the study did not include direct patient involvement, ethical clearance was not obtained.

Under RNCTP, category I includes new patients with smear-positive pulmonary tuberculosis, new patients with severe forms of extrapulmonary tuberculosis and smear-negative pulmonary tuberculosis. Category II includes patients who had been previously treated for tuberculosis and require re-treatment. This group includes patients with relapse, treatment failure and defaulters. Category III includes new patients with smear negative tuberculosis and extrapulmonary tuberculosis who are not seriously ill. The intensive phase of treatment is for two months in categories I and III; and for three months, in category II. The continuation phase is for four months in category I and II; and for five months, in category II. The sputum smear examination is done at the end of two months in category I; and at the end of three months, in category II. If the sputum smear remains positive during follow-up, the intensive phase is continued for one more month. The sputum smear is done again at 3, 4 and 7 months in category I; and at 4, 6 and 9 months, in category II.

Default was defined as patients not taking anti-TB drugs for two months or more, consecutively after starting treatment.[12] Default rate was calculated as the total number of patients who defaulted in each group divided by the total number of patients initiated on treatment in that particular group and was expressed as a proportion. The Kaplan-Meier curve was drawn to make an overall estimate of patients continuing treatment at the end of each month in the three DOTS categories. The difference in the patterns of default over time between different groups was studied using the log rank test.

Analysis was done to study the time of default in smear-positive patients. The time of default was ascertained by calculating the difference between the date of initiation and date of outcome. In all patients who had defaulted, the date on which the patients missed the treatment for the first time was considered as the date of outcome, i.e., default. The difference was divided by 30 to decide the month in which the event occurred. For example, if the difference was 100 days, it was divided by 30 to get the number 3.33, which was inferred as ‘outcome (default) occurred in the fourth month.’ The events which were censored were death, treatment completion, cure, failure and transfer-out. Probability of default in each month was calculated. The cumulative probability of default by the end of each month was calculated by adding the default rate in the particular month and the cumulative default rate in the previous month.

The sputum smear conversion and treatment outcome were also assessed with Fisher exact test to understand the implications of the time of default. Cure and treatment completion were considered as favorable outcomes, while default, death and failure were considered as unfavorable outcomes.

## RESULTS

A total of 716 patients were registered at the Tuberculosis Unit during the year 2004. Of these, 275 belonged to category I; 106, to category II; and 335, to category III. Overall, the default rate was 10.33% (74/716).

**Figure 1:**
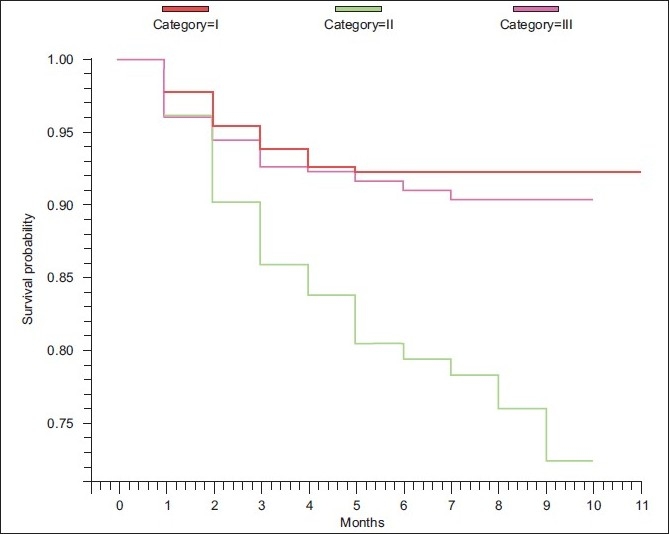
Kaplan-Meier plot of the default over time after initiation of treatment in the three DOTS categories

Figure 1 illustrates the Kaplan-Meier analysis of default over time after initiation of treatment in the three DOTS categories. The cumulative default rates by the end of two months were 4%, 10% and 5% in the categories I, II and III, respectively. The cumulative default rates at the end of the treatment period were 8%, 27% and 9% in the three categories, respectively. There was a significant difference in the default rate over time between the patients in the three DOTS categories (log rank statistic= 15.49; d.f.= 2; *P*=0.0004). In the first month of treatment, the default rates were similar in categories II and III, i.e., 4%. During the rest of the treatment period, the cumulative default rate was consistently higher in category II as compared to that in categories I and III. The dips seen in category II in Figure 1 indicate the higher default rates in this group throughout the treatment.

A total of 331 patients had smear-positive pulmonary tuberculosis. Amongst the 95 patients with smear-positive tuberculosis in category II, 56 (59%) were patients put on re-treatment after default; 16 (17%), as a result of failure of previous treatment; and 23 (24%) had relapse. The default rate was 30.35% (17/56) in re-treatment after default; 12.5% (2/16), in re-treatment after failure; and 13% (3/23), in re-treatment after relapse.

Table 1 lists the default rates over various periods (in months) after initiation of treatment amongst smear-positive patients in category I and category II. In category I, maximum default is seen in the first month of treatment, i.e., 2.11%. The cumulative default rate at the end of two months was 3.91%; and at the end of six months, 6.23%. The default rate at the end of the eighth month, when all patients were censored, was also 6.23%. In category II, maximum default (8.89%) occurred in the second month, followed by third month (5%). The cumulative default rate by the end of second month was 10.99%; by the end of the third month, 16%; and by the end of eighth month, 26%. The default rate by the end of the tenth month, by which time all patients were censored, was 30.89%.

**Table 1:** Default over various periods (in months) after treatment initiation in the smear-positive patients on DOTS

DOTS Category	Months	Number of patients initiated on treatment	Number of defaulters	Number of patients censored	Probability of default in the month	Cumulative probability of default by the end of the month
Category I	1	236	5	8	2.11	2.11
	2	223	4	1	1.79	3.91
	3	218	3	3	1.37	5.28
	4	212	2	4	0.94	6.23
	5	206	0	5	0.00	6.23
	6	201	0	73	0.00	6.23
	>6	128	0	128	0.00	6.23
Category II	1	95	2	3	2.11	2.11
	2	90	8	3	8.89	10.99
	3	79	4	0	5.06	16.06
	4	75	2	1	2.67	18.72
	5	72	2	1	2.78	21.50
	6	69	0	2	0.00	21.50
	7	67	2	4	2.99	24.49
	8	61	1	39	1.64	26.13
	>8	21	1	20	4.76	30.89

Of the total default, 64% in category I and category II occurred by the end of the intensive phase of treatment. Only two patients, both in category I, defaulted within the first two weeks of treatment.

Of the total 74 patients who defaulted, 11 patients had extrapulmonary tuberculosis, 27 had smear-negative pulmonary tuberculosis and 36 patients had smear-positive tuberculosis at initiation of treatment.

Table 2 lists the treatment outcomes based on the results of the sputum smear examination at the end of intensive phase. The risk of unfavorable outcome was significantly more in patients in whom sputum smear was positive (28/35) compared to patients who tested negative (12/263) at the end of intensive phase (*P*<0.0001). The cure/ treatment completion rate was high (84.52%) in patients who had smear-negative results at the end of intensive phase. The patients who had persistent smear-positive results at the end of intensive phase of treatment had high failure rates (55.56%). Sputum smear could not be done in patients who transferred out, defaulted or died in the intensive phase or immediately after completion of intensive phase of treatment. Amongst those who defaulted in continuation phase, six patients had persistent sputum smear positivity at the end of the intensive phase; and in seven patients, sputum smear tested negative at the end of the intensive phase of treatment.

**Table 2:** Sputum conversion at the end of intensive phase and treatment outcome

Sputum conversion at the end of intensive phase	Total number of patients	Treatment outcome				
		Cured/Treatment completed	Default	Death	Failure	Transferred out
Sputum smear negative	253	214 (84.58)	7 (2.77)	5 (1.98)	0 (0)	0 (0)
Sputum smear positive	36	6 (16.67)	6 (16.67)	2 (5.56)	20 (55.56)	0 (0)
Sputum smear not done	42	0 (0)	23 (54.76)	17 (40.48)	0 (0)	2 (4.76)
Total	331	219 (66.36)	36 (10.91)	24 (7.27)	20 (6.06)	2 (0.61)

## DISCUSSION

There is limited understanding of the timing of default in patients on treatment of tuberculosis in the developing world. In our study, the default rates of patients in categories I, II and III at the end of the intensive phase were 4%, 14% and 5%, respectively. Amongst the smear-positive patients, 64% of the total default occurred in the intensive phase of treatment. Default began in the first month after treatment initiation and continued till the last month of treatment in category II.

A wide range of default times has been reported from other studies and reflects the differences in period of study, context, patients and specific programs. In a study from India, default started in the third month and increased up to the fourth month and subsequently declined.[9] In a study from Bangalore city in India, 65.7% of the default in category I and 71% of default in category II were reported in the intensive phase.[13] In another study in south India, 53 (72%) out of 74 smear-positive patients in category I defaulted during intensive phase of treatment.[10] In a study from Zambia, 29.8% of the patients under DOTS stopped taking their medication within the first two months of commencing treatment.[14] In a study of default conducted in Tashkent, Uzbekistan, it was reported that patients defaulted mostly during the intensive phase.[15] Other studies have reported higher proportions of default in the continuation phase of treatment.[16–18]

The treatment under DOTS is given in two phases. The initial intensive phase of treatment is designed to kill actively growing and semi-dormant bacilli and is intended to shorten the duration of infectiousness with rapid smear conversion after two to three months of treatment. The use of four to five drug regimens in the intensive phase reduces the risk of development of drug resistance, failure and relapse. The continuation phase eliminates most residual bacilli and reduces failure and relapses. At the start of the continuation phase, the numbers of bacilli are expected to be low with less chance of selecting drug-resistant mutants.[19]

Studies have reported sputum smear conversion rates at the end of the intensive phase to be in the range of 60% to 85%.[20,21] The culture conversion at the end of intensive phase has been observed in 88% of the patients.[20]

In a study, sputum conversion rate at the end of the first month was 71%; at the end of the second month, 84%; and at the end of the third month, 92%.[21] A significant decrease in conversion rate has been observed with initial high-grade smear positive status.[20–22]

Poor compliance with treatment is also an important factor in the development of acquired drug resistance. In a study, treatment after default was found to be the most important factor associated with drug resistance, as 45 of the 48 isolates from patients with history of default showed drug resistance.[23] The implications of default will depend on the time of default. The earlier a patient defaults, more will be the chances of persistent smear-positive status and risk of drug resistance.

Patients who are smear positive at the end of intensive phase have an increased risk of failure. It has been reported that a significant number of patients who remain positive at the end of intensive phase fail treatment.[4,24]

Hence patients who default in the intensive phase of treatment and with persistent smear positivity at the end of intensive phase should be retrieved for continuing treatment on a priority basis.

Another important finding of the study is the higher rates of default throughout the treatment duration in category II patients. A high default rate has been reported in the re-treatment group in the RNTCP reports.[25,26] More than half of the category II consists of patients requiring re-treatment after default. These patients have more risk of default during re-treatment. Treatment after default has been identified as a risk factor for default.[27–29] Studies done on default have described many risk factors and reasons for default. These issues are not addressed routinely in RNTCP. Hence when patients return for re-treatment, the problems persist, increasing the risk of repeat default. In case of failure and relapse, the patients have already completed 5-6 month regimens in the recent past and are expected to take treatment for another 8 months’ period. The outlook of the patients towards the long duration of treatment needs to be assessed.

It is important to correlate the reasons of default with the time of default. This will help to focus on specific issues in different phases of treatment to prevent default. It is suggested that the RNTCP reports should include the time of default in the routine reports, especially the data regarding the number of patients who default before completing the intensive phase of treatment.
